# Ferroptosis‐Driven Neuronal Damage Exacerbation in Diabetic Stroke: Implications of SLC7A11 Inhibition

**DOI:** 10.1002/cns.70830

**Published:** 2026-03-12

**Authors:** Siyuan Liu, Min Liu, Binbin Wang, Miao Sun, Huikai Yang, Mengyao Qu, Libin Ma, Likai Shi, Yuxiang Song, Peng Li, Lulu Zhou, Wugang Hou, Weidong Mi, Yulong Ma

**Affiliations:** ^1^ Department of Anesthesiology Affiliated Hospital of Nantong University Nantong China; ^2^ Department of Anesthesiology The First Medical Center of Chinese PLA General Hospital Beijing China; ^3^ Department of Anesthesiology Beijing Tongren Hospital, Capital Medical University Beijing China; ^4^ Department of Anesthesiology The Sixth Medical Center of Chinese PLA General Hospital Beijing China; ^5^ Department of Oncology The First Affiliated Hospital of Jinzhou Medical University Jinzhou Liaoning Province China

**Keywords:** diabetes mellitus, ferroptosis, ischemic stroke, SLC7A11

## Abstract

**Background:**

Diabetes mellitus is a known contributor to worsened neurological outcomes following ischemic stroke, yet the underlying mechanisms remain elusive. Emerging evidence links ferroptosis to the extent of neuronal damage postischemic stroke. However, the role of ferroptosis in the context of diabetic stroke remains uncharted. SLC7A11, a key player in cellular antioxidant defense and ferroptosis regulation, has unexplored functions in diabetic cerebral ischemia/reperfusion.

**Methods:**

In this study, we employed leptin receptor deficient (db/db) mice and wild‐type (WT) littermates to model cerebral ischemic stroke. We harnessed quantitative proteomics to identify differentially expressed proteins and uncover potential signaling pathways. Infarct volume was assessed via 2,3,5‐triphenyltetrazolium chloride staining. Neurobehavioral tests, immunohistochemistry, and immunofluorescence staining were employed to evaluate neuronal injury. The expression of SLC7A11 was assessed using Western blot and immunofluorescence staining. Dihydroethidium staining, flow cytometry, immunofluorescence staining, transmission electron microscopy, immunohistochemistry, and ELISA were used to assess ferroptosis. Furthermore, an adeno‐associated viral 9 (AAV9) vector encoding SLC7A11 was utilized to validate the protective potential of neuronal SLC7A11 overexpression during diabetic stroke.

**Results:**

Diabetic stroke mice exhibited exacerbated cerebral injury, with differentially expressed proteins notably enriched in ferroptosis. Ferroptosis inhibitors markedly ameliorated neuronal damage in diabetic stroke mice. Additionally, we uncovered inhibited SLC7A11 expression and heightened ferroptosis in the early stages of reperfusion during diabetic stroke. Neuron‐specific enforced expression of SLC7A11 mitigated neuronal lipid peroxidation and ferroptosis, providing neuroprotective effects against diabetic cerebral ischemic injury. These protective effects were underscored by the decreased infarct volume, enhanced neurological function, and increased neuronal survival.

**Conclusion:**

Our study points to SLC7A11 inhibition‐mediated ferroptosis as a pivotal factor in the aggravated neuronal damage observed during diabetic stroke. This novel insight opens avenues for potential therapeutic targets in the management of diabetic stroke, focusing on interventions aimed at modulating ferroptosis or SLC7A11 expression.

## Graphical abstract

The current study uncovered aggravated cerebral ischemic injury and neurological dysfunction following diabetic stroke, in which ferroptosis was involved. SLC7A11 expression was inhibited in the early stages of reperfusion during diabetic stroke, and both ferroptosis inhibitors and neuron‐specific enforced expression of SLC7A11 mitigated neuronal lipid peroxidation and ferroptosis during diabetic stroke, providing neuroprotective effects against diabetic cerebral ischemic injury.

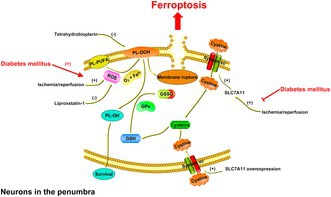

**Abbreviations**
AAV: Adeno‐associated viral
ACSL4: Acyl‐CoA synthetase long‐chain family member 4
BH4: Tetrahydrobiopterin
CNS: Central nervous system
CP: Ceruloplasmin
DCF: 2′,7′‐dichlorofluorescein
DCFH: 2′,7′‐dichlorodihydrofluorescein
DHE: Dihydroethidium
DM: Diabetes mellitus
4‐HNE: 4‐hydroxynonenal
Ftmt: Mitochondrial ferritin
GO: Gene Ontology
GPX4: Glutathione peroxidase 4
HE: Hematoxylin and Eosin
HO‐1: Heme oxygenase‐1
I/R: Ischemia/reperfusion
KEGG: Kyoto Encyclopedia of Genes and Genomes
Lip‐1: Liproxstatin‐1
MCA: Middle cerebral artery
MCAO: Middle cerebral artery occlusion
MDA: Malondialdehyde
MFI: Mean fluorescence intensity
ROS: reactive oxygen species
SOCS2: suppressor of cytokine signaling 2
TEM: Transmission electron microscopy
TMT: Tandem mass tag
TTC: 2,3,5‐triphenyltetrazolium chloride

## 1 Background

Cerebral stroke is a debilitating ailment, often culminating in fatality or enduring incapacitation, with nearly 90% of cases being ischemic [1]. One of the predominant risk factors associated with ischemic stroke is diabetes mellitus (DM), a condition that amplifies the risk of stroke by as much as threefold [2]. Furthermore, individuals afflicted with diabetes manifest deteriorated neurological function and a higher incidence of disability following stroke events [3]. Experimental models involving diabetic animals have likewise exhibited worsened neurological outcomes after ischemic stroke [4, 5]. Nonetheless, the precise underlying mechanisms behind the heightened cerebral injury observed in diabetic stroke remain largely enigmatic.

Ferroptosis is a form of cell death dependent on iron and ignited by lipid peroxidation, often triggered by the accumulation of reactive oxygen species (ROS) following system xc^−^ inhibition [6, 7]. Previous studies have unveiled the involvement of ferroptosis in various diabetic complications, including diabetic nephropathy, diabetic cardiomyopathy, and diabetic retinopathy, primarily attributable to diminished antioxidant capacity and elevated lipid peroxide levels [8, 9, 10]. Furthermore, ferroptosis has been implicated in the pathogenesis of several neurological conditions such as Alzheimer's disease [11], Parkinson's disease [12] and traumatic brain injury [13]. Previous findings have suggested a positive correlation between neuronal ferroptosis and the extent of neuronal damage following cerebral ischemia [14, 15]. However, whether ferroptosis plays a role in the aggravated cerebral injury during diabetic stroke remains uncertain.

The cystine‐glutamate antiporter system xc^−^ is pivotal in facilitating cystine uptake, essential for preserving cellular antioxidant activity, and providing neuroprotection against oxidative stress within the central nervous system (CNS) [16, 17, 18]. System xc^−^ encompasses two key components, SLC7A11 and SLC3A2, with SLC7A11 being stress‐inducible and serving as a critical defense protein in response to oxidative stress, while SLC3A2 is expressed constitutively [19, 20]. SLC7A11 holds a pivotal role in maintaining cellular redox homeostasis and acts as a central regulator of ferroptosis [21, 22]. Inhibition of SLC7A11 hampers cellular antioxidant defenses, exacerbates ROS accumulation, accelerates lipid peroxidation, and triggers ferroptosis [23]. Nevertheless, it remains unclear whether SLC7A11 contributes to the enhanced neuronal losses observed during diabetic stroke.

To investigate these questions, we employed leptin receptor deficient (db/db) mice, characterized by spontaneous obesity, hyperglycemia and insulin resistance, as a well‐established rodent model for type 2 DM [24]. In the current study, we utilized db/db mice and their phenotypically normal littermates (WT) to establish a middle cerebral artery occlusion (MCAO)/reperfusion (MCAO/R) model. We compared the extent of neuronal injury between db/db mice and WT mice, employing quantitative proteomic analysis to identify differentially expressed proteins and unravel associated signaling pathways. Subsequently, we examined the involvement of ferroptosis and the expression of SLC7A11 in the exacerbation of cerebral injury during diabetic stroke. Finally, we introduced an AAV9 viral vector encoding SLC7A11 into the diabetic mice to confirm the impact of neuronal SLC7A11 during diabetic stroke. Our study endeavors to shed light on a potential novel mechanism underpinning the aggravated cerebral injury in diabetic stroke, with implications for the therapeutic potential of ferroptotic inhibition in this pathogenesis.

## 2 Materials and Methods

### 2.1 Animals

Male adult db/db mice and WT mice were purchased from GemPharmatech (Nanjing, China). The mice were aged between 6 and 8 weeks and weighed 35–45 g and 20–25 g, respectively. The animals were housed in a controlled environment with a standard temperature (23°C ± 2°C) and maintained on a 12–12 h light–dark cycle. They were provided with ad libitum access to water and food. The experiments were performed after 2 weeks of acclimatization to the laboratory conditions. Body weight and fasting blood glucose levels of db/db mice are exhibited in Figure S1. All experimental procedures were approved by the Ethics Committee for Animal Experimentation of Chinese PLA General Hospital and were performed in accordance with relevant ethical guidelines.

### 2.2 Middle Cerebral Artery Occlusion (MCAO) and Reperfusion (MCAO/R)

Mice were put in an induction chamber filled with 3% isoflurane for anesthesia induction, and then maintained with isoflurane at an initial concentration of 2%, adjusted according to interdigital reflex. A heating pad was used to maintain rectal temperatures at 37°C ± 0.5°C during the surgical procedure. Following exposure of the right common carotid artery (CCA), a silicone‐coated monofilament was inserted to the origin of the middle cerebral artery (MCA) via the CCA. The MCAO model was established in both the Con + MCAO and db/db + MCAO groups. The same surgery procedure was performed in the Con + Sham and db/db + Sham groups except for the insertion of monofilament. The MCA was occluded for 60 min, and the monofilament was subsequently removed to initiate reperfusion. Laser Doppler flowmetry (RWD Life Science CO. LTD, China) was utilized to ascertain cerebral blood flow to the MCA territory, ensuring confirmation of occlusion and reperfusion (Figure S2). Only mice demonstrating a minimum 75% reduction in cortical cerebral blood flow and at least 75% recovery to the baseline following reperfusion were included in the study.

### 2.3 Drug Treatment

The db/db mice were subjected to ferroptosis inhibitor treatment. Liproxstatin‐1 (10 mg/kg; MedChem‐Express, USA) and tetrahydrobiopterin (10 mg/kg; MedChem‐Express, USA) or an equivalent volume of vehicle (10% DMSO in PBS, Beyotime, China) were intraperitoneally administered to mice once daily for five consecutive days. MCAO was performed immediately after the last injection and the subsequent experiments were conducted by researchers blinded to the treatment.

### 2.4 Infarct Volume Assessment

Infarct volume was assessed using 2,3,5‐triphenyltetrazolium chloride (TTC, Sigma‐Aldrich, USA) staining. Mice were euthanized by cervical decapitation after being deeply anesthetized by isoflurane. Fresh whole brains were excised and sliced into six 1‐mm‐thick coronal sections located 1 mm from the frontal pole. Brain slices were immersed in a 2% TTC solution in phosphate buffered saline at 37°C for 30 min and then fixed in 4% paraformaldehyde (Servicebio, China) at 4°C for 24 h. The infarct area was quantified using Adobe Photoshop (version 22.4). Relative infarct volumes were determined as follows: contralateral brain tissue areas minus non‐infarcted areas of the ipsilateral brain tissue, divided by the contralateral regional volume and multiplied by 100 as a percent of the contralateral, correcting for both cerebral edema and body weight. The total relative infarct volume was calculated as the sum of the relative infarct volumes from all six slices.

### 2.5 Neurological Deficits Evaluation

Neurological deficits were assessed using the modified Garcia test, foot fault test, and the corner task. The modified Garcia test evaluated neurological deficits from the following aspects: forelimb walking, symmetry of limb movement, whisker stimulation, climbing and lateral turning [25]. The foot fault test recorded 50 steps for each mouse, with falls or slips during the test recorded as foot faults [26]. The corner task evaluated the integrated sensorimotor function regarding sensory neglect and motor response. Mice were placed between two cardboards attached at an angle of 30. With the cardboards gradually approaching, the mice reared upward when the vibrissae were stimulated, and turned back to face the opening. The direction in which the animals turned around was recorded from ten trials for each mouse, with movements without rearing not scored [27].

### 2.6 Proteomic Analysis

Ischemic penumbra cortex tissues were grinded using liquid nitrogen and urea (with protease inhibitor cocktail) was used for sonication. Following centrifugation, the supernatant was collected, and the protein concentrations were determined using a BCA Protein Assay reagent kit (Solarbio life sciences, China). The protein solution was reduced with dithiothreitol, alkylated with iodoacetamide, and then diluted by adding 100 mM TEAB to urea concentration less than 2 M. Finally, trypsin was added at a 1:50 trypsin‐to‐protein mass ratio for an initial overnight digestion, followed by a 1:100 trypsin‐to‐protein mass ratio for a subsequent 4‐h digestion. Peptides were TMT labeled using a TMT kit (ThermoFisher Scientific, USA) and fractionated into segments through high‐pH reverse‐phase HPLC (ThermoFisher Scientific, USA) according to the manufacturer's protocol. The peptides were subjected to tandem mass spectrometry (MS/MS) in Q ExactiveTM Plus (ThermoFisher Scientific, USA). Proteins with log2 fold change > 1.3 and *p* < 0.05 are defined as upregulated, while proteins with log2 fold change < 1/1.3 and *p* < 0.05 are defined as downregulated. Gene Ontology (GO) annotation proteome was derived from the UniProt‐GOA database (http://www.ebi.ac.uk/GOA/). Identified protein domain functional description were annotated by InterProScan and the InterPro domain database (http://www.ebi.ac.uk/interpro/) was used. Kyoto Encyclopedia of Genes and Genomes (KEGG) database (http://www.genome.jp/kegg/) was used to annotate protein pathway, and cluster analysis of pathways was performed using Metascape (https://metascape.org).

### 2.7 Malondialdehyde (MDA) and 4‐Hydroxynonenal (4‐HNE) Assay

Ischemic penumbra cortex tissues were homogenized in RIPA lysis buffer containing protease inhibitor and phosphatase inhibitor cocktail on ice to extract total proteins. Total protein concentrations were determined using a BCA Protein Assay reagent kit (Solarbio life sciences, China) according to the manufacturer's instructions. Levels of MDA and 4‐HNE were measured using the MDA Assay Kit (Nanjing Jiancheng, China) and the 4‐HNE Assay Kit (ab238538, Abcam, UK), in accordance with the manufacturer's protocols. Results for MDA and 4‐HNE were expressed as nanomoles per milligram of total protein (nmol/mg prot) and micrograms per milligram of total protein (μg/mg prot), respectively.

### 2.8 Reduced Glutathione (GSH)/Oxidized Glutathione (GSSG) Ratio and Iron Assay

Protein samples were prepared as described for the MDA assay. GSH and GSSG levels were measured using GSH (AKPR008C, Boxbio Science & Technology, China) and GSSG (AKPR009C, Boxbio Science & Technology, China) Assay Kits according to the manufacturer's instructions. The GSH/GSSG ratio was calculated according to the GSH and GSSG levels. Ferrous iron (Fe^2+^) levels were determined using the iron assay kit (ab83366, Abcam, UK) in accordance with the manufacturer's protocols. Results for Fe^2+^ content were expressed as nanomoles per milligram of total protein (nmol/mg prot).

### 2.9 Hematoxylin and Eosin (HE) Staining, Nissl Staining

Mice were deeply anesthetized and were transcardially perfused with PBS, followed by 4% paraformaldehyde. Brains were dissected out and fixed in 4% paraformaldehyde overnight. Paraffin‐embedded, the brains were coronally cut into 4‐μm‐thick slices. HE staining and Nissl staining were performed in strict accordance with the manufacturer's manuals of the HE staining kit (Servicebio, China) and Nissl staining kit (Servicebio, China).

### 2.10 Western Blot Analysis

Protein samples were prepared as described for the MDA assay. An equal amount of protein sample (20 μg) was separated by 4%–20% SDS–PAGE and then transferred onto a PVDF membrane. The membranes were blocked with 5% BSA and then incubated overnight at 4°C with primary antibodies. The primary antibodies used in the current study were rabbit anti‐xCT (1:1000, ab175186, Abcam, UK), rabbit anti‐FACL4 (1:500, ab155282, Abcam, UK), rabbit anti‐GPX4 (1:1000, cst59735, Cell Signaling Technology, USA), and mouse anti‐β‐actin (1:1000, ab125066, Abcam, UK). Following three washes with TBST buffer, the membranes were then incubated with the corresponding HRP conjugated secondary antibody for 1 h at room temperature. Immunoblots were visualized and quantified using a chemiluminescence system. Relative protein levels were normalized to β‐actin, serving as a loading control for total protein.

### 2.11 Immunofluorescence Staining

Coronal sections were prepared in the same manner as for HE staining. Primary antibodies used for immunofluorescence staining included rabbit anti‐SLC7A11 (1:100, PA5‐116134, Invitrogen, USA), mouse anti‐4‐Hydroxynonenal (1:50, MA5‐27570, Invitrogen, USA), rabbit anti‐NeuN (1:200, cst24307, Cell Signaling Technology, USA), and mouse anti‐NeuN (1:200, ab104224, Abcam, UK). Sections were incubated with mixtures of Alexa‐488 (green, Invitrogen, USA) or Alexa‐594 (red, Invitrogen, USA)‐conjugated donkey anti‐rabbit or donkey anti‐mouse secondary antibodies for 2 h for double staining at room temperature.

### 2.12 Dihydroethidium (DHE) Staining

ROS levels were assessed via DHE staining. Fresh brains were isolated, frozen, and then cut into coronal sections with a cryostat. DHE dye (2 μmol/L, Servicebio, China) was applied to the frozen sections, followed by incubation in a light‐protected, humidified chamber at 37°C for 30 min and subsequent washing with PBS. An observer blinded to group allocation randomly selected six different fields of view for each section, and mean fluorescence intensity (MFI) was analyzed using ImageJ software.

### 2.13 Flow Cytometry for ROS Detection

Fresh brain tissues from the ischemic penumbra were prepared as above. After enzymatic digestion, the brain tissues were dissociated using the gentleMACS Octo Dissociator (Miltenyi Biotec, Germany), and single‐cell suspensions were prepared. We used ROS‐sensitive probe 2′,7′‐dichlorodihydrofluorescein (DCFH‐DA, Beyotime, China) to detect intracellular ROS levels according to the manufacturer's protocols. The intracellular ROS was able to oxidize DCFH to generate 2′,7′‐dichlorofluorescein (DCF), and the fluorescence intensity of DCF was detected by flow cytometry.

### 2.14 Transmission Electron Microscopy (TEM)

Fresh tissue specimens from the ischemic penumbra were cut into 2 mm^3^ cubes and fixed in 2.5% glutaraldehyde (Servicebio, China) for 2 h at room temperature, followed by overnight fixation at 4°C. An osmium tetroxide postfixation was applied to stabilize the sample. The samples were then dehydrated in 70%, 90%, and 100% acetone, and subsequently sectioned at a thickness of 80 nm for further observation. Mitochondria around the nucleus in neurons were observed, and the number of shrunken mitochondria accompanied by increased membrane density and disappeared cristae was recorded.

### 2.15 Adeno‐Associated Viral (AAV) Administration

AAV9 viral particles containing hSyn‐MCS‐WPRE for neuronal‐specific SLC7A11 overexpression, or an empty vector, were purchased from Shanghai Genomeditech. Stereotaxic AAV injection was performed on db/db mice using a Hamilton micro‐syringe. Mice were anesthetized and placed on the stereotaxic apparatus, with the following stereotaxic coordinates relative to bregma: site 1, anterior 0.5 mm; lateral 1.5 mm; dorsoventral 3.5 mm; site 2, posterior 0.3 mm; lateral 2.5 mm; dorsoventral 3.3 mm; site 3, posterior 2 mm; lateral 1.5 mm; dorsoventral 3.3 mm^27^. A volume of 0.7 μL of AAV9 (1 × 10^13^ genomic copies) was injected at each site at a flow rate of 0.15 μL/min, and the needle was held in place for 5 min after injection. MCAO was performed 4 weeks following injection.

### 2.16 Statistical Analysis

Data were analyzed using GraphPad Prism 9.0 software (GraphPad, San Diego, CA, USA) and results were presented as mean ± SEM. ImageJ was utilized to process the density of specific bands and fluorescence intensity. Differences between two groups were analyzed using student's *t*‐test, and multiple group comparisons were analyzed via one‐way ANOVA followed by Bonferroni correction for the multiple comparisons. A value of *p* < 0.05 was considered statistically significant.

## 3 Results

### 3.1 Aggravated Cerebral Ischemic Injury and Neurological Dysfunction Following Diabetic Stroke

**FIGURE 1:**
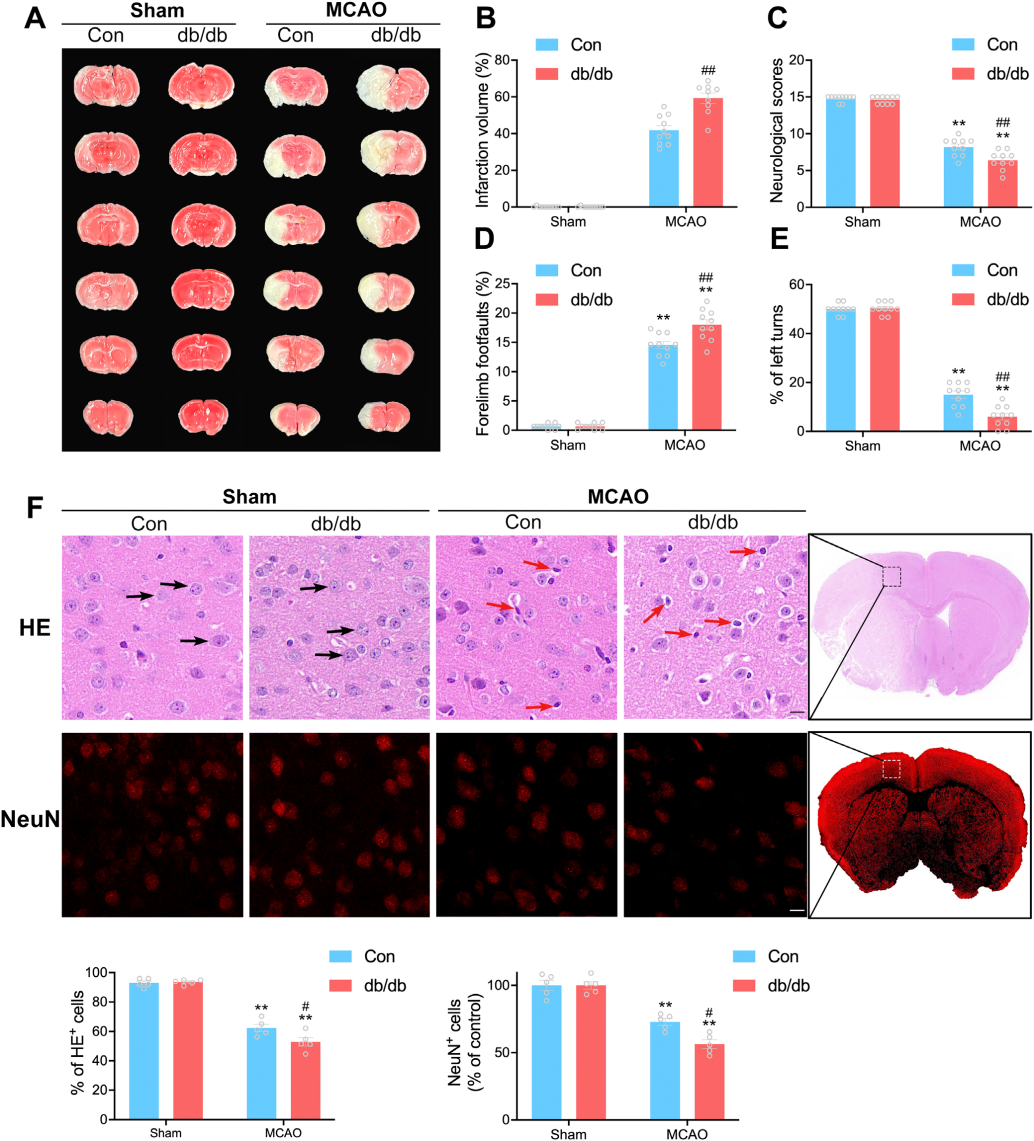
Aggravated cerebral ischemic injury and neurological dysfunction following diabetic stroke. (A) Representative TTC‐stained brain sections at 24 h post‐reperfusion. (B) Quantification and analysis of the infarct volume (*n* = 9–10/group). (C–E) Neurological functions were assessed by modified Garcia score test (C), foot fault test (D) and corner test (E) (*n* = 10/group). (F) Representative images and quantification of HE staining and NeuN staining at 24 h post‐reperfusion in the ischemic penumbra (*n* = 5/group). Black arrows denote compact cell outline. Red arrows denote pyknotic nuclei. Scale bars = 10 μm. Differences between two groups were analyzed using student's *t*‐test, and multiple group comparisons were analyzed via one‐way ANOVA followed by Bonferroni correction for the multiple comparisons. *p < 0.05, **p < 0.01 vs. corresponding Sham. ^#^ *p* < 0.05, ^##^ *p* < 0.01 versus Con + MCAO. Data are presented as mean ± SEM.

To comprehensively evaluate the extent of neuronal damage following diabetic stroke, we subjected db/db mice and WT (Con) mice to 1‐h MCAO. Infarct volumes and neurological function were assessed after 24 h of reperfusion. The db/db + MCAO group exhibited significantly increased infarct volumes (%) compared to the Con + MCAO group (59.23 ± 2.82 vs. 41.82 ± 2.49, ^##^ *p* < 0.01, Figure 1A,B). Furthermore, the deterioration in neurological function was evident in the db/db + MCAO group, as indicated by reduced neurological scores, increased forelimb foot faults, and fewer left turns (Figure 1C–E).

HE staining and NeuN staining were employed to assess neuronal losses within the ischemic penumbra. As revealed in Figure 1F, HE staining depicted consistent brain tissue staining and intact nucleoli (HE^+^) in both non‐diabetic sham and diabetic sham groups. The proportion of HE^+^ cells in the ischemic penumbra declined after MCAO, with a more pronounced effect observed in the db/db + MCAO group compared to the Con + MCAO group (52.87 ± 2.84 vs. 62.37 ± 2.44, ^#^ *p* < 0.05). NeuN staining, indicating surviving neurons after cerebral ischemia, showed a decrease in the proportion of NeuN‐positive cells in both non‐diabetic and diabetic groups after MCAO. The db/db + MCAO group displayed more severe neuronal loss in comparison to the Con + MCAO group (56.35 ± 3.24 vs. 72.83 ± 2.57, ^#^ *p* < 0.05). These results provide robust confirmation that cerebral injury and neurological dysfunction are exacerbated following diabetic stroke.

### 3.2 Ferroptosis Is Involved in Aggravated Cerebral Injury After Diabetic Stroke

**FIGURE 2:**
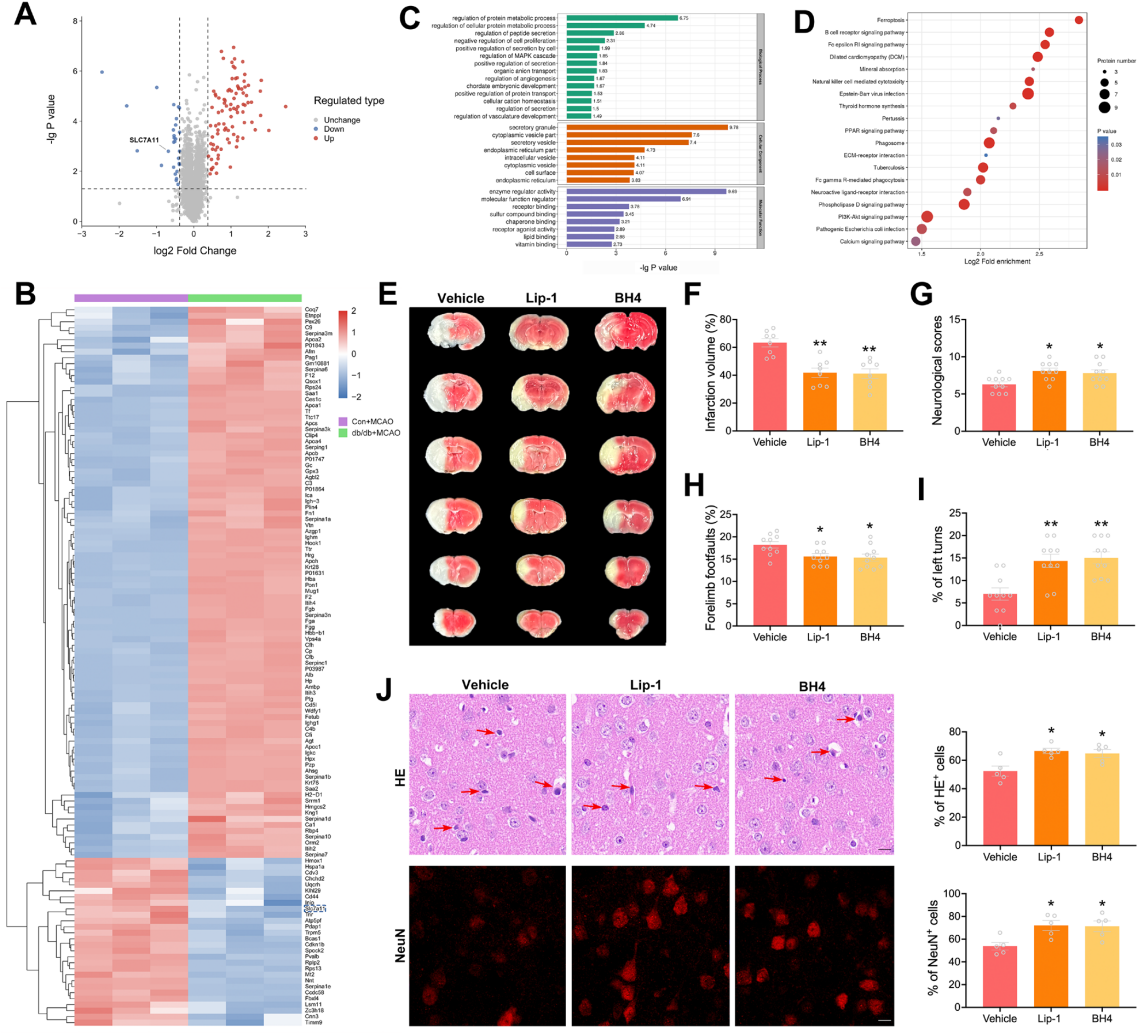
Ferroptosis is involved in aggravated cerebral injury after diabetic stroke. (A) Volcano plot of differentially expressed proteins in db/db + MCAO group relative to Con + MCAO group (*n* = 3/group). Upregulated proteins with log2 fold change > 1.3 and *p* < 0.05 are shown in red. Downregulated proteins with log2 fold change < 1/1.3 and *p* < 0.05 are shown in blue. (B) Heat map of 120 differentially expressed proteins. (C) Gene ontology analysis of differentially expressed proteins. The x‐axis represents −lg(*p* value). (D) Kyoto Encyclopedia of Genes and Genomes analysis of differentially expressed proteins. The x‐axis represents log2 fold enrichment. (E) Representative TTC‐stained brain sections of db/db mice treated with vehicle or ferroptosis inhibitors at 24 h post‐reperfusion. (F) Quantification and analysis of the infarct volume (*n* = 8/group). (G–I) Neurological functions were assessed by modified Garcia score test (G), foot fault test (H) and corner test (I) (*n* = 8/group). (J) Representative images and quantification of HE staining and NeuN staining at 24 h post‐reperfusion in the ischemic penumbra. Red arrows denote pyknotic nuclei (*n* = 5/group). Scale bars = 10 μm. Statistical analysis was performed using one‐way ANOVA followed by Bonferroni correction for the multiple comparisons. *p < 0.05, **p < 0.01 versus Vehicle group. Data are presented as mean ± SEM.

To uncover the molecular mechanisms contributing to the aggravated cerebral injury in diabetic stroke, a tandem mass tag labeled quantitative proteomic analysis was conducted using brain tissues from the ischemic penumbra at 24 h post‐reperfusion. This analysis identified a total of 5439 proteins, of which 5030 were quantifiable. Notably, 92 upregulated proteins and 28 downregulated proteins were detected in diabetic MCAO mice (Figure 2A,B). GO analysis revealed that the most enriched biological process was the regulation of protein metabolism, the most enriched cellular component was the secretory granule, and the most enriched molecular function was enzyme regulator activity (Figure 2C). KEGG pathway analysis indicated that the differentially expressed proteins were significantly enriched in pathways related to ferroptosis, B cell receptor signaling pathway, and Fc epsilon receptor‐I signaling pathway (Figure 2D).

Of particular interest was the involvement of ferroptosis, a recently defined cell death process driven by unhinged lipid peroxidation. Differentially expressed proteins related to ferroptosis included ceruloplasmin (CP), transferrin (TF), SLC7A11, and heme oxygenase‐1 (HO‐1). To validate the role of ferroptosis in diabetic stroke, ferroptosis inhibitors, Liproxstatin‐1 (Lip‐1) and tetrahydrobiopterin (BH4), were administered to diabetic mice followed by MCAO. The results from TTC staining confirmed that both Lip‐1 and BH4 treatment significantly reduced infarct volumes in diabetic stroke mice (Figure 2E,F). Moreover, these inhibitors ameliorated neurological deficits, as evidenced by improved neurological scores, fewer forelimb foot faults, and increased left turns (Figure 2G–I). Importantly, neuronal survival in the ischemic penumbra was rescued by the administration of ferroptosis inhibitors, as demonstrated by increased HE^+^ cells and NeuN^+^ cells (Figure 2J). These results emphasize the role of ferroptosis in the aggravated cerebral injury during diabetic stroke.

### 3.3 Deficient SLC7A11 Expression During Diabetic Stroke

**FIGURE 3:**
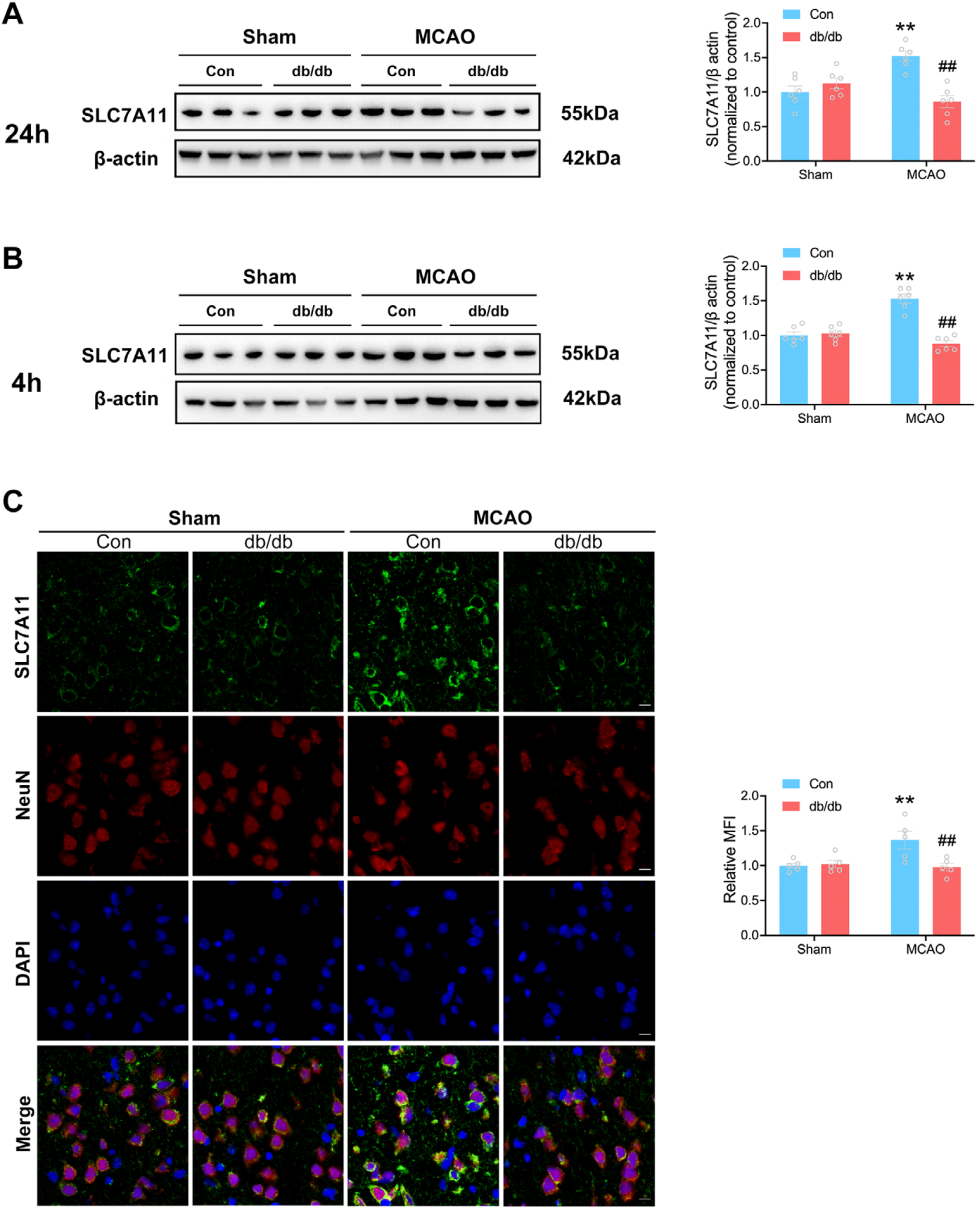
Deficient SLC7A11 expression during diabetic stroke. (A) Representative immunoblot and quantification of SLC7A11 protein levels at 24 h post‐reperfusion in the penumbra, β‐Actin was used as internal control (*n* = 6/group). (B) Representative immunoblot and quantification of SLC7A11 protein levels at 4 h post‐reperfusion in the penumbra, β‐Actin was used as internal control (*n* = 6/group). (C) Representative immunofluorescence images and quantification of the mean fluorescence intensity (MFI) of SLC7A11 in the ischemic penumbra at 4 h post‐reperfusion (*n* = 5/group). Scale bars = 10 μm. Statistical analysis was performed using one‐way ANOVA followed by Bonferroni correction for the multiple comparisons. **p < 0.01 versus corresponding Sham. ^##^ *p* < 0.01 versus Con + MCAO. Data are presented as mean ± SEM.

The proteomic analysis also revealed the marked downregulation of SLC7A11 in diabetic stroke mice, suggesting impaired antioxidant capacity. To validate these findings, SLC7A11 protein levels were examined at 24 h post‐reperfusion. A significant increase in SLC7A11 expression was observed in non‐diabetic mice following cerebral ischemia/reperfusion (I/R). However, this upregulation was not observed in diabetic mice (Figure 3A). As oxidative stress occurs rapidly after reperfusion, it was crucial to determine if cerebral I/R induced SLC7A11 upregulation at early time points. Figure 3B shows that SLC7A11 upregulation was evident in non‐diabetic stroke mice at 4 h post‐reperfusion, while diabetic stroke mice did not exhibit this response. Furthermore, immunofluorescence staining with a neuron marker (NeuN) confirmed upregulation of SLC7A11 in neurons of the ischemic penumbra 4 h post‐reperfusion in the Con + MCAO group, but not in the db/db + MCAO group (Figure 3C). These results suggest that cerebral I/R induced neuronal SLC7A11 upregulation, but the expression of SLC7A11 was deficient during diabetic stroke, which suggested an early antioxidant mechanism deficiency in diabetic stroke animals.

### 3.4 Exacerbated Oxidative Stress and Neuronal Lipid Peroxidation During Diabetic Cerebral I/R

**FIGURE 4:**
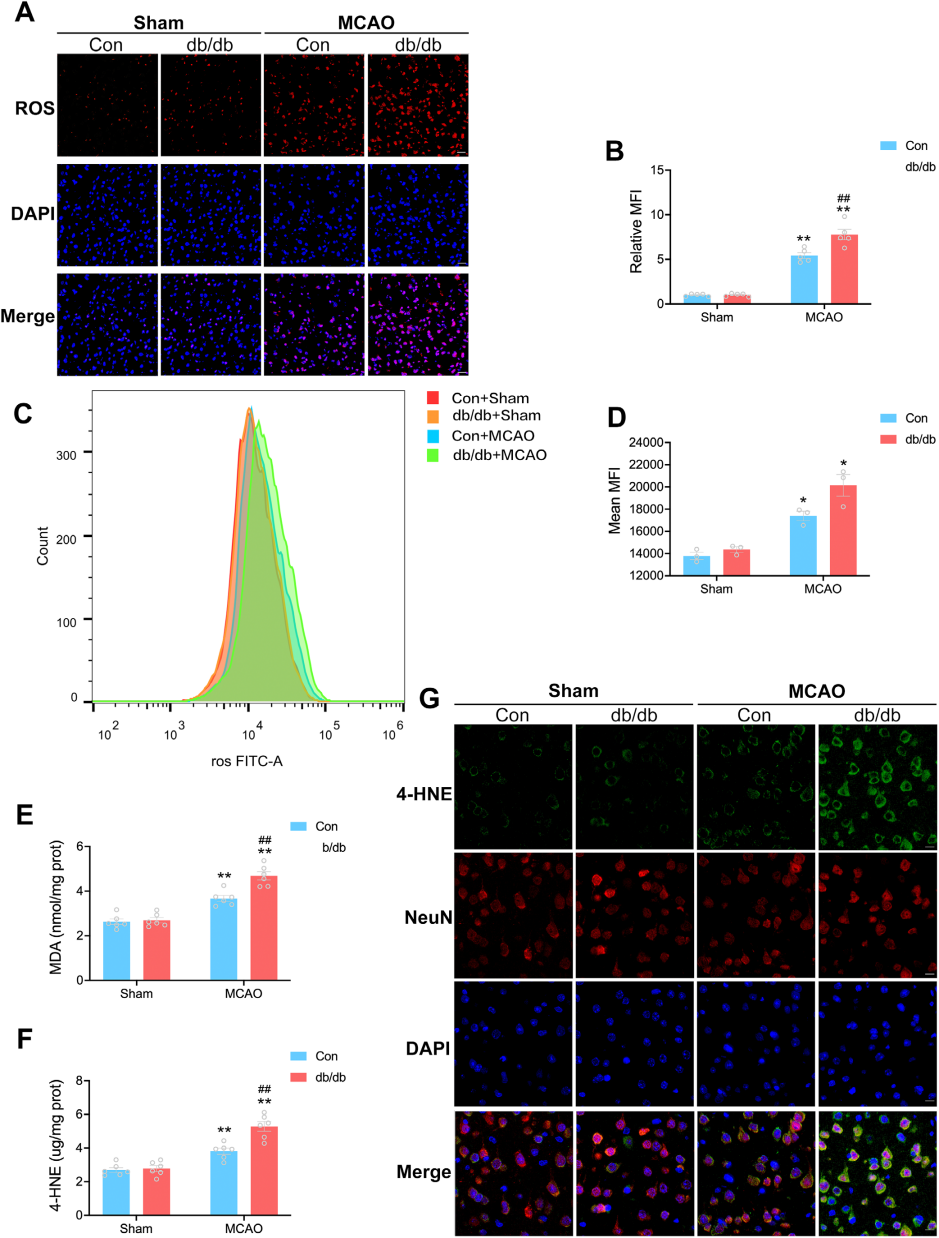
Exacerbated oxidative stress and neuronal lipid peroxidation during diabetic cerebral I/R. (A) Representative images of Dihydroethidium (DHE) staining at 4 h post‐reperfusion, which represents ROS levels in the ischemic penumbra. Scale bars = 25 μm. (B) Quantification of the mean fluorescence intensity (MFI) of ROS (*n* = 5/group). (C) ROS levels were measured by the MFI of 2′,7′‐dichlorofluorescein (DCF) by flow cytometry. (D) Quantification of the MFI of DCF (*n* = 3/group). (E and F) MDA and 4‐HNE levels in the ischemic penumbra at 4 h post‐reperfusion (*n* = 6/group). (G) Representative immunofluorescence images of 4‐HNE in the ischemic penumbra at 4 h post‐reperfusion. Scale bars = 10 μm. Statistical analysis was performed using one‐way ANOVA followed by Bonferroni correction for the multiple comparisons. *p < 0.05, **p < 0.01 versus corresponding Sham. ^##^ *p* < 0.01 versus Con + MCAO. Data are presented as mean ± SEM.

SLC7A11 upregulation protects neurons from oxidative stress and subsequent lipid peroxidation [28, 29]. Having revealed that the forced expression of SLC7A11 in the ischemic penumbra was inhibited at the early phase of cerebral I/R during diabetic stroke, we then asked whether oxidative stress and lipid peroxidation were also accelerated during this phase. DHE staining and flow cytometry were used to evaluate ROS levels in the ischemic penumbra at 4 h post‐reperfusion. DHE staining revealed that ROS production was elevated in the ischemic penumbra following reperfusion in both the Con + MCAO group and db/db + MCAO group, but ROS production was more pronounced in the db/db + MCAO group (Figure 4A,B). We then qualified ROS levels using flow cytometry, which detected ROS in living cells via a ROS‐sensitive probe. It was also observed in flow cytometry that the db/db + MCAO group exhibited much higher levels of ROS compared to the Con + MCAO group (Figure 4C,D). Furthermore, the levels of MDA and 4‐HNE, crucial end‐products of lipid peroxidation, were measured at 4 h post‐reperfusion. Both MDA (nmol/mg prot) and 4‐HNE (μg/mg prot) contents increased following cerebral I/R, with the db/db + MCAO group exhibiting a greater increase in both markers compared to the Con + MCAO group (4.68 ± 0.19 vs. 3.67 ± 0.14, ^##^ *p* < 0.01, 5.29 ± 0.28 vs. 3.82 ± 0.19, ^##^ *p* < 0.01, respectively, Figure 4E,F).

Considering that neurons are particularly vulnerable to high levels of peroxidative stress resulting from excessive ROS, double immunofluorescent staining was used to evaluate neuronal lipid peroxidation, a driving force of ferroptosis. Results of double immunofluorescent staining showed that a majority of neurons were 4‐HNE‐positive following cerebral I/R (Figure 4G). These findings collectively suggest that oxidative stress and neuronal lipid peroxidation were exacerbated in the ischemic penumbra during the early phase of diabetic cerebral I/R.

### 3.5 Enhanced Neuronal Ferroptosis and Neuronal Losses During Diabetic Stroke

**FIGURE 5:**
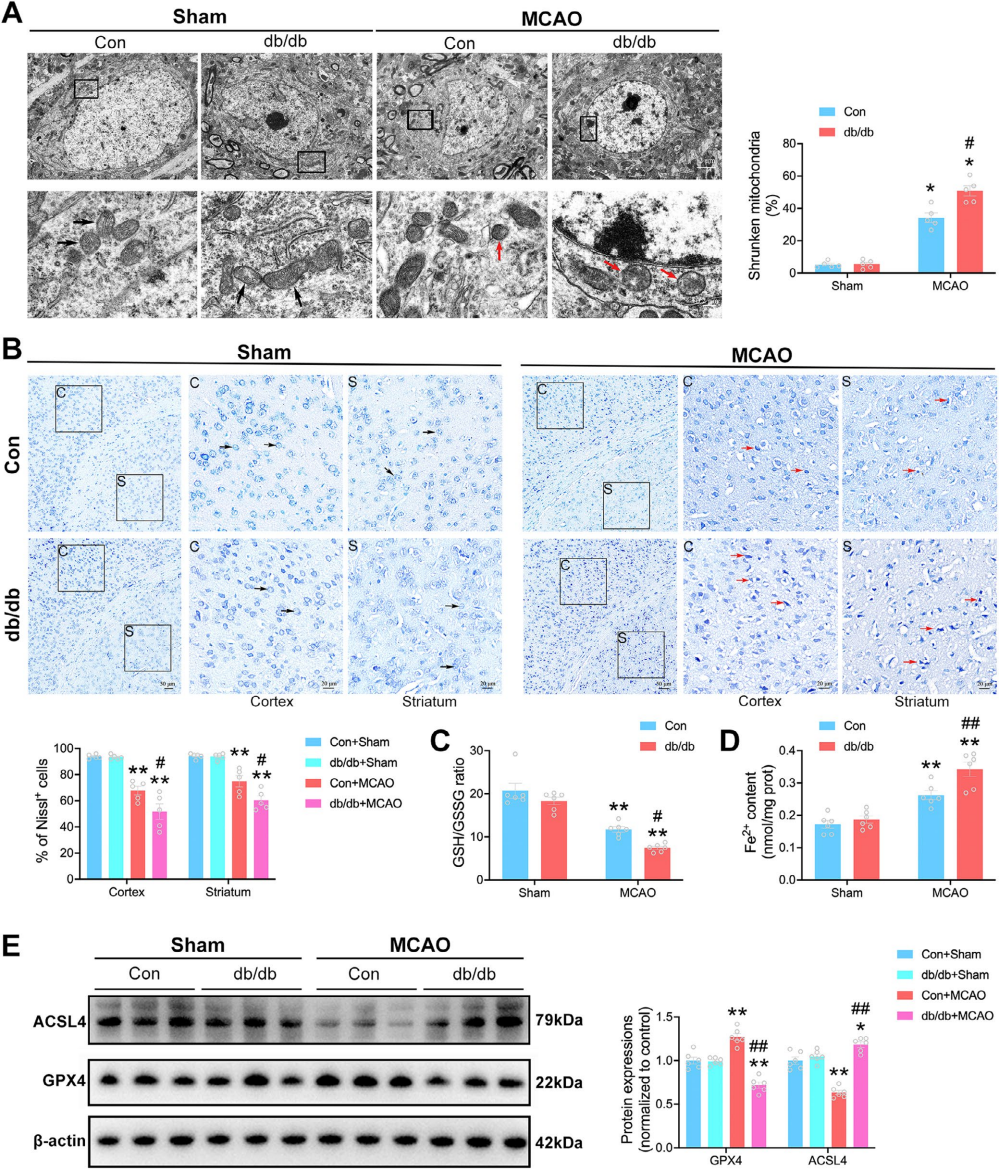
Enhanced neuronal ferroptosis and neuronal losses during diabetic stroke. (A) Representative transmission electron microscopy images and quantification of morphologically altered neuronal mitochondria in the ischemic penumbra (*n* = 5/group). The characteristic morphological change of ferroptosis was the shrunken mitochondrial accompanied by increased membrane density and disappeared crista. Black arrows denote elongated mitochondria with intact crista. Red arrows denote shrunken mitochondria with disappeared crista. (B) Representative images and quantification of Nissl staining that indicated morphological neuronal alterations in cortex and striatum in the ischemic penumbra (*n* = 5/group). C represents cortex and S represents striatum. Black arrows denote neurons with flush Nissl granules. Red arrows denote degenerated neurons with reduced Nissl granules. (C) GSH/GSSG ratio in the ischemic penumbra at 4 h post‐reperfusion (*n* = 6/group). (D) Fe^2+^ levels in the ischemic penumbra at 4 h post‐reperfusion (*n* = 6/group). (E) Representative immunoblot and quantification of ACSL4 and GPX4 protein expression in the penumbra (*n* = 6/group). Statistical analysis was performed using one‐way ANOVA followed by Bonferroni correction for the multiple comparisons. *p < 0.05, **p < 0.01 versus corresponding Sham. ^#^ *p* < 0.05, ^##^ *p* < 0.01 versus Con + MCAO. Data are presented as mean ± SEM.

Accumulation of ROS and lipid peroxides is well‐established hallmarks of ferroptosis. To explore whether neuronal ferroptosis was indeed enhanced in the ischemic penumbra during the early phase of reperfusion in diabetic stroke, TEM was utilized to analyze mitochondrial ultrastructure in neurons at 4 h post‐reperfusion. Abnormally altered mitochondria were observed in both the Con + MCAO group and db/db + MCAO group. Importantly, I/R‐induced mitochondrial damage was more pronounced in the diabetic stroke mice, as indicated by a greater proportion of shrunken mitochondria (%) in the db/db + MCAO group compared to the Con + MCAO group (50.81 ± 3.18 vs. 34.19 ± 2.92, ^#^ *p* < 0.05, Figure 5A).

Next, we performed Nissl staining to assess neuronal morphological alterations. Neurons in both Con+Sham and db/db + Sham groups exhibited a clear outline, compact structure, and flush cytoplasmic compartment following Nissl staining. However, Nissl‐positive neurons were found to be decreased after MCAO in both the cortex and striatum within the penumbra, with the reduction being more pronounced in the db/db + MCAO group (Figure 5B).

Indicators related to ferroptosis such as GSH/GSSG ratio, Fe^2+^ levels, ACSL4 and GPX4 expression were also evaluated. GSH/GSSG ratio decreased while Fe^2+^ levels elevated after cerebral ischemia in both the Con + MCAO group and db/db + MCAO group. GSH/GSSG ratio was significantly lower in the diabetic mice than those in non‐diabetic mice after cerebral ischemia, whereas Fe^2+^ levels showed the opposite trend (Figure 5C,D). ACSL4 expression was downregulated while GPX4 was upregulated after cerebral ischemia in the Con + MCAO group. The protein expression levels of ACSL4 in the db/db + MCAO group were higher than those in the Con + MCAO group, whereas GPX4 expression was lower than those in the Con + MCAO group (Figure 5E). These findings underline the aggravation of neuronal ferroptosis in the early phase of cerebral I/R during diabetic stroke, which, in turn, exacerbates neuronal injury and leads to greater neuronal losses within the ischemic penumbra.

### 3.6 Neuron‐Specific SLC7A11 Overexpression Alleviated Early‐Triggered Neuronal Ferroptosis During Diabetic Stroke

**FIGURE 6:**
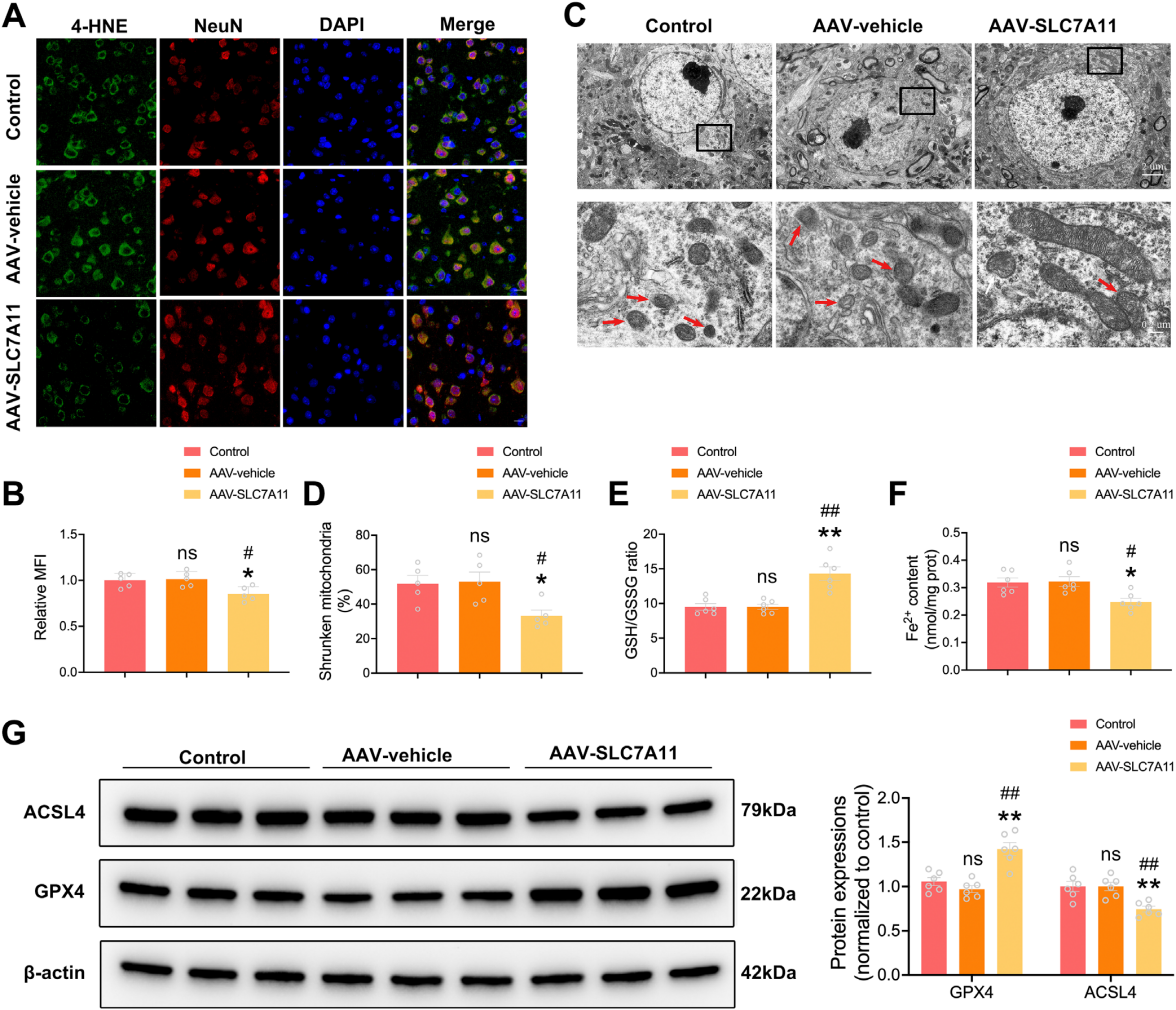
SLC7A11 overexpression in neurons alleviated early‐triggered neuronal ferroptosis during diabetic stroke. (A) Representative immunofluorescence images of 4‐HNE in the ischemic penumbra at 4 h post‐reperfusion. (B) Quantification of the mean fluorescence intensity (MFI) of 4‐HNE. (*n* = 5/group). Scale bars = 10 μm. (C) Representative transmission electron microscopy images of morphologically altered neuronal mitochondria in the ischemic penumbra. The characteristic morphological change of ferroptosis was the shrunken mitochondrial accompanied by increased membrane density and disappeared crista. Red arrows denote shrunken mitochondria with disappeared crista. (D) Quantification of morphologically altered neuronal mitochondria (*n* = 5/group). (E) GSH/GSSG ratio in the ischemic penumbra at 4 h post‐reperfusion (*n* = 6/group). (F) Fe^2+^ levels in the ischemic penumbra at 4 h post‐reperfusion (*n* = 6/group). (G) Representative immunoblot and quantification of ACSL4 and GPX4 protein expression in the penumbra (*n* = 6/group). Statistical analysis was performed using one‐way ANOVA followed by Bonferroni correction for the multiple comparisons. *p < 0.05, **p < 0.01 versus control, ^#^ *p* < 0.05, ^##^ *p* < 0.01 versus AAV‐vehicle, ns indicates nonsignificant. Data are presented as mean ± SEM.

The collective evidence points toward SLC7A11 as a crucial player in diabetic stroke pathophysiology. To test whether upregulated SLC7A11 can render neurons resistant to ferroptosis during diabetic cerebral I/R, an AAV9 vector encoding SLC7A11 under the control of the synapsin promoter was employed in diabetic mice. Western blot and double immunofluorescent staining confirmed the significant upregulation of SLC7A11 protein levels in the AAV‐SLC7A11 group at 4 h following cerebral I/R (Figure S3). Double immunofluorescent staining revealed that neuronal 4‐HNE expression was decreased in the AAV‐SLC7A11 group in comparison to both the control group and AAV‐vehicle group (Figure 6A,B). SLC7A11 overexpression also alleviated mitochondrial pathological alterations following diabetic cerebral I/R, as evidenced by a reduced proportion of shrunken mitochondria in the AAV‐SLC7A11 group (Figure 6C,D). After cerebral ischemia, GSH/GSSG ratio increased and the levels of Fe^2+^ decreased with SLC7A11 overexpression in the diabetic mice (Figure 6E,F). Additionally, ACSL4 expression was downregulated while GPX4 was upregulated after SLC7A11 overexpression in the diabetic stroke mice (Figure 6G). Taken together, these results revealed that SLC7A11 overexpression alleviated ferroptosis during diabetic cerebral I/R.

### 3.7 Neuron‐Specific SLC7A11 Overexpression Reduced Cerebral Damage Following Diabetic Stroke

**FIGURE 7:**
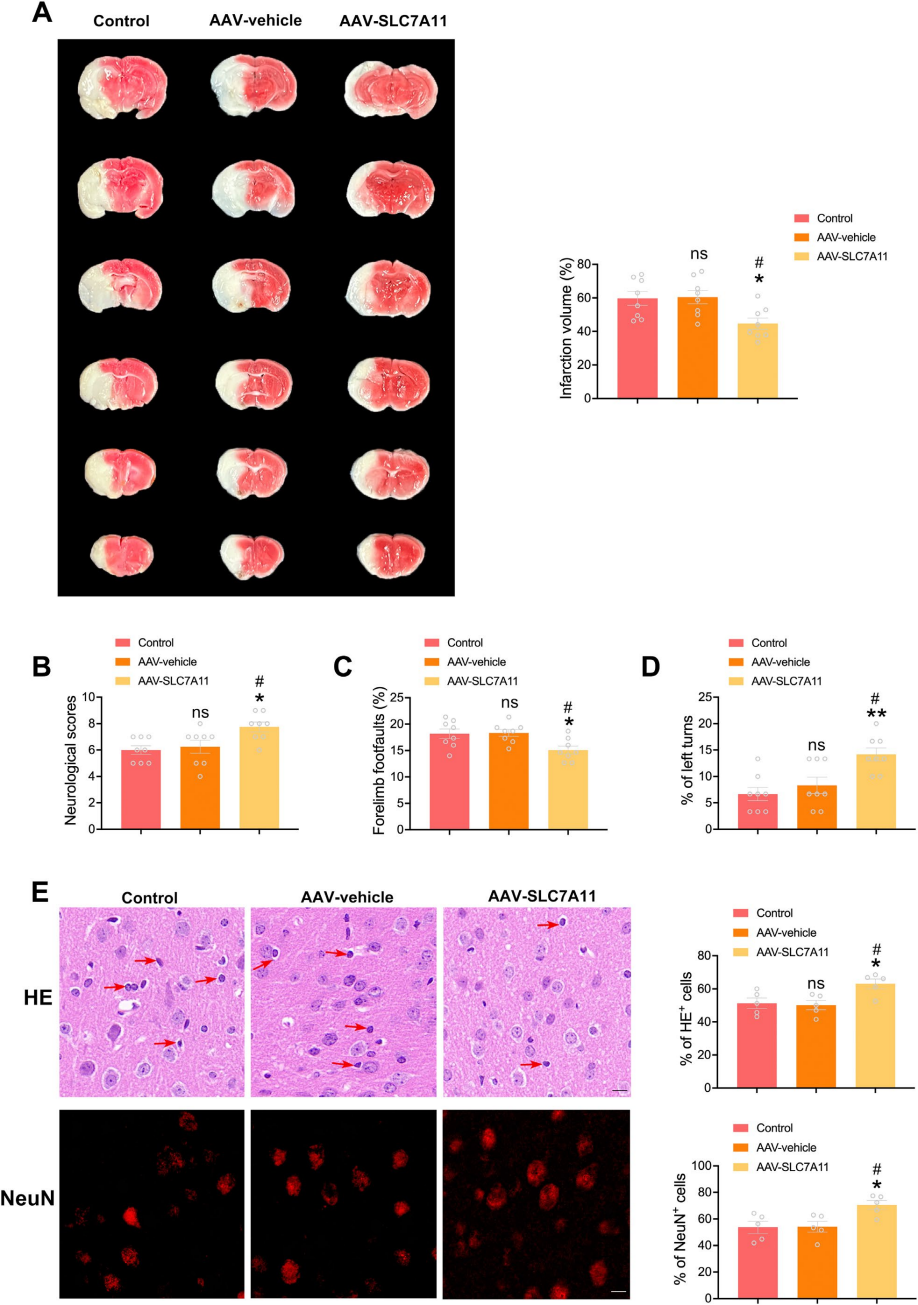
SLC7A11 overexpression in neurons reduced cerebral damage following diabetic stroke. (A) Representative images and quantification of TTC staining (*n* = 8/group). (B–D) Neurological functions were assessed by modified Garcia score test (B), foot fault test (C) and corner test (D) (*n* = 8/group). (E) Representative images and quantification of HE staining and NeuN staining (*n* = 5/group). Red arrows denote pyknotic nuclei. Scale bars = 10 μm. Statistical analysis was performed using one‐way ANOVA followed by Bonferroni correction for the multiple comparisons. *p < 0.05, **p < 0.01 versus control, ^#^ *p* < 0.05 versus AAV‐vehicle, ns indicates nonsignificant. Data are presented as mean ± SEM.

The potential therapeutic impact of neuronal SLC7A11 overexpression was assessed through TTC staining to measure infarct volumes. While the infarct volumes of the control group and AAV‐vehicle group were similar, diabetic mice injected with AAV‐SLC7A11 displayed significantly reduced infarct volumes (Figure 7A). Moreover, compared to the control group and AAV‐vehicle group, neurological function was notably improved in the AAV‐SLC7A11 group, with higher neurological scores, fewer forelimb foot faults, and increased left turns (Figure 7B–D). A concomitant increase in the proportion of HE^+^ cells and NeuN‐positive cells within the ischemic penumbra was evident in the AAV‐SLC7A11 group in comparison to the control group and AAV‐vehicle group (Figure 7E). These findings collectively highlight the efficacy of neuronal SLC7A11 overexpression in mitigating cerebral damage and preserving neuronal survival in the ischemic penumbra after diabetic stroke.

In summary, these results collectively underscore the impact of diabetic stroke, showcasing the contribution of deficient SLC7A11‐mediated antioxidant defense, enhanced ferroptosis, and increased neuronal losses. Importantly, the targeted overexpression of SLC7A11 has been found to effectively alleviate early‐triggered neuronal ferroptosis and reduce cerebral damage, offering a promising avenue for therapeutic intervention in the context of diabetic stroke.

## 4 Discussion

Diabetic stroke patients frequently face challenges in achieving favorable functional recovery even when recanalization rates are comparable to non‐diabetic stroke patients [30, 31]. This study aimed to elucidate the molecular mechanisms underpinning the exacerbated neuronal injury during diabetic cerebral I/R. Our findings reaffirmed that diabetic mice exhibit heightened cerebral injury and neurological dysfunction after reperfusion following MCAO, consistent with prior studies [4, 5]. Ferroptosis, a form of cell death initiated by overwhelming lipid peroxidation, emerged as a central player in the pathophysiology of ischemic stroke [14, 15]. This study provides new insights into the pivotal role ferroptosis plays in the progression of neuronal injury during diabetic cerebral I/R. Importantly, our results highlight potential interventions, such as ferroptosis inhibitors and the modulation of SLC7A11, which may hold therapeutic promise in treating diabetic stroke by curbing neuronal ferroptosis.

The ischemic penumbra, a region partially deprived of blood supply but potentially viable with the restoration of normal perfusion, has become a critical target for ischemic stroke research [32]. Our quantitative proteomic analysis of the ischemic penumbra unveiled significant changes in protein expression profiles. Notably, the differentially expressed proteins were enriched in processes related to ferroptosis. This is a significant finding, as ferroptosis has recently gained recognition as a critical component of cerebral I/R injury [14, 15].

To validate the involvement of ferroptosis in diabetic stroke, we employed ferroptosis inhibitors Lip‐1 and BH4 in our experimental model. Both Lip‐1 and BH4 inhibit ferroptosis independent of the glutathione antioxidant system. Lip‐1 is a validated ferroptosis inhibitor with great capacity to trap peroxyl radicals in lipid bilayers and subvert ferroptosis [33]. BH4 is a potent antioxidant that can diffuse through bilayer membranes to eliminate lipid peroxidation [34]. These interventions led to significantly reduced infarct volumes, improved neurological function, and enhanced neuronal survival. The effectiveness of ferroptosis inhibition underscores the crucial role of ferroptosis in exacerbating neuronal damage in diabetic stroke.

Our proteomic analysis revealed a marked difference in SLC7A11 expression between non‐diabetic and diabetic stroke mice. SLC7A11 has been previously recognized as a crucial defense mechanism against oxidative species within the CNS^16^. Enhanced SLC7A11 expression under oxidative stress conditions typically augments cellular antioxidant capacity, restores redox homeostasis, and promotes neuronal survival [35]. Elevated glutamate induced a prominent upregulation of SLC7A11 in HT22 cells, which also rendered the neurons resistant to hydrogen peroxide [36]. Contrastingly, decreased SLC7A11 expression induced lipid ROS overproduction, promoted neuronal ferroptosis in an epileptic mouse model [28] and was involved in neuronal ferroptosis during amyotrophic lateral sclerosis [37]. Our results corroborate this hypothesis of SLC7A11, the key regulator of ferroptosis, being upregulated in the early phase of cerebral I/R in the penumbra, which is consistent with a plethora of studies highlighting the neuroprotective properties of SLC7A11 in various stress conditions [20, 35, 38]. However, our research identifies a novel deficiency in the adaptive SLC7A11 upregulation during diabetic stroke. The early suppression of SLC7A11 activity in diabetic mice, intended to counteract ferroptosis and protect neurons, emerges as a significant contributing factor to neuronal losses during diabetic cerebral I/R. This deficiency tips the balance toward increased vulnerability to ferroptosis, exacerbating neuronal injury.

Neuronal injury following ischemic stroke is primarily attributable to interrupted cerebral blood supply and subsequent I/R injury. The restoration of oxidative phosphorylation during cerebral I/R leads to excessive ROS production [39], which gives rise to the antioxidant signal activation to reacquire redox homeostasis [40]. The penumbral neurons are particularly vulnerable to adverse events that may lead to metabolic disruption, especially the excessive production of ROS. Indeed, our study reveals that diabetic stroke mice exhibit accelerated ROS production and lipid peroxidation in the ischemic penumbra shortly after reperfusion. This heightened oxidative stress in diabetic stroke coincides with previous research indicating that hyperglycemia increases neuronal superoxide production after cerebral ischemia [41]. This accelerated oxidative stress is a driving force behind lipid peroxidation, a process that generates toxic lipid ROS and aggravates neuronal injury during cerebral I/R [42]. In particular, we highlight the critical role of inadequate repair of lipid peroxides, leading to uncontrolled lipid peroxide propagation, membrane damage, and ferroptosis initiation.

Acyl‐CoA synthetase long‐chain family member 4 (ACSL4) determines cellular sensitivity to ferroptosis and is a key regulator of ferroptosis induction [43]. Cui Y et al. demonstrated that ACSL4 overexpression through lentivirus injection aggravated neuronal damage during ischemic stroke, while ACSL4 knockdown alleviated ischemic brain injury [15]. Glutathione peroxidase 4 (GPX4) is one of the negative regulators of ferroptosis that maintains cellular redox homeostasis [44]. Living cells obtain cysteine primarily through SLC7A11‐mediated uptake of extracellular cystine, followed by the reduction of intracellular cystine to cysteine, which is subsequently utilized for glutathione biosynthesis. The state of the GSH/GSSG ratio serves as a key indicator of cellular redox homeostasis and correlates with sensitivity to oxidative stress. Additionally, GSH is a cofactor for GPX4 to detoxify lipid peroxidation [45]. In the current research, downregulation of ACSL4 and upregulation of GPX4 in the ischemic penumbra were observed, suggesting an adaptive response to ferroptotic stimuli following ischemic stroke. However, ferroptosis inevitably occurred during the pathology of stroke, evidenced by enhanced oxidative stress and lipid peroxidation, mitochondrial pathological alterations, decreased GSH/GSSG ratio, and elevated Fe^2+^ levels. It is worth noting that the diabetic stroke mice exhibited the opposite trend in ACSL4 and GPX4 expression, indicating a dysfunction in ferroptosis regulation following ischemic stroke. Besides, the diabetic stroke mice also exhibited enhanced neuronal ferroptosis phenotype compared to the non‐diabetic stroke mice. Previous investigations have shown that enhanced neuronal ferroptosis was related to aggravated neuronal injury following cerebral I/R, and diabetes worsened I/R injury during stroke [46, 47]. Our results are consistent with those of the previous studies. Our findings provide the first evidence that neuronal ferroptosis was enhanced in the ischemic penumbra shortly after reperfusion during diabetic stroke and indicate the potential for interventions targeting this pathway.

In the present research, we observed that the adaptive SLC7A11 upregulation was inhibited since the early phase of cerebral I/R. We also validated that enhanced SLC7A11 expression inhibited ferroptosis and alleviated neuronal injury following cerebral ischemic in diabetic mice. Reduced serum antioxidant defense capacity, increased lipid peroxidation, and ferroptosis‐related molecules deregulation have been reported in diabetic patients [48, 49]. Li W et al. reported that ferroptosis was involved in aggravated myocardial injury during diabetic myocardial I/R in a rat model [50]. Lin Y et al. discovered that osteoblastic ferroptosis was activated in diabetic osteoporosis, during which decreased SLC7A11 expression was involved [51]. Inhibition of ferroptosis through Fer‐1 treatment repaired antioxidant capacity and reduced lipid peroxidation, while SLC7A11 expression was decreased in a diabetic nephropathy mouse model [52]. Disruption of the cystine/SLC7A11/glutathione axis was also observed in a diabetic Parkinson's disease (PD) model, resulting in accelerated neuronal degeneration and PD susceptibility [38]. However, no studies correlated the antioxidant defense deficiency or ferroptosis with reperfusion‐related processes during diabetic stroke. Here we report early deficiency of SLC7A11 activity in diabetic stroke mice, which suggests a role in the neuronal losses in diabetic cerebral I/R. In addition, we also validated the role of SLC7A11 in neuronal ferroptosis and the role ferroptosis plays in the subsequent neuronal injury during diabetic stroke. These findings hold promise for the development of novel therapeutic strategies for diabetic stroke. Neuronal death following cerebral ischemia is the predominant cause of neurological dysfunction after stroke. Considering that the greater neuronal loss caused by enhanced ferroptosis will result in the conversion of hypo‐perfused brain tissue into infarction and will aggravate brain injury during ischemic stroke, interventions aimed at slowing down the penumbral neuronal ferroptotic cell death represent an exciting avenue for potential treatments. By targeting the early phases of ferroptosis, we may mitigate neuronal damage, improve cerebral injury outcomes, and enhance neurological function in diabetic stroke patients.

While our study provides valuable insights into the role of ferroptosis and SLC7A11 in diabetic stroke, it is important to acknowledge certain limitations. The specific molecular mechanism by which SLC7A11 was regulated during diabetic cerebral I/R was not elucidated in the present study. Previous research discovered that *SLC7A11* was downregulated by p53 [53], while the RNA‐binding protein RBMS1 was a post‐translation regulator of SLC7A11 expression [54]. The suppressor of cytokine signaling 2 (SOCS2) was also identified as an SLC7A11‐interacting protein facilitating degradation through ubiquitination [55]. Regulation of SLC7A11 function under ferroptotic stress during diabetic stroke might be multifactorial, and future research should delve deeper into the precise molecular mechanisms governing SLC7A11 regulation during diabetic cerebral I/R, ultimately offering a more comprehensive understanding of this emerging therapeutic target. The translation of these insights to clinical applications has the potential for groundbreaking advancements in the management of diabetic stroke, ultimately improving the lives of those affected by this challenging condition.

## 5 Conclusion

This study advances our understanding of the mechanisms underlying exacerbated cerebral injury in diabetic stroke, shedding light on the critical role of neuronal ferroptosis in the early phase of cerebral I/R. Our results provide compelling evidence that the adaptive upregulation of SLC7A11, a key regulator of ferroptosis and oxidative stress defense, is impaired in the context of diabetic ischemic stroke. Since SLC7A11 inhibition‐mediated ferroptosis could be a potential mechanism relevant to the aggravated cerebral injury in diabetic stroke, treatments slowing down the penumbral neuronal ferroptotic cell death might be of help in diabetic stroke treatment.

## Author Contributions

Y.M., S.L., and W.M. designed the experiments. S.L., M.L., B.W., W.H., W.M., and Y.M. conducted data analysis, drafted and revised the manuscript. S.L. and M.L. participated in animal model construction. M.Q., Y.H., L.M., L.S., Y.S., and P.L. were responsible for mice grouping and behavioral tests. S.L., M.L., B.W., M.S., and H.Y. performed experiments.

## Funding

This study was financially supported by the National Natural Science Foundation of China (No. 82371469, 82171464, 81801193, and 82501755), the Natural Science Foundation of Beijing (No. 7254318), and Jiangsu Provincial Research Hospital Fund (YJXYY202204‐YSC07).

## Ethics Statement

The study protocol was approved by the Ethics Committee for Animal Experimentation of Chinese PLA General Hospital (reference number: 2022‐X18‐93).

## Conflicts of Interest

The authors declare no conflicts of interest.

## Supporting information


**Figure S1:** Body weight and fasting blood glucose in db/db mice. (A) Body weight in db/db mice; (B) Fasting blood glucose in db/db mice.

**Figure S2:** Representative images of laser speckle flowmetry. Only the mice with at least 75% decline of cortical cerebral blood flow and 75% recovery of the baseline after reperfusion were included for the study.

**Figure S3:** Representative immunoblot and immunofluorescent staining of SLC7A11 protein expression. (A) Representative immunoblot and quantification of SLC7A11 protein levels at 4 h post‐reperfusion in the penumbra, β‐actin was used as internal control. SLC7A11 was upregulated in the AAV‐SLC7A11 group compared to the control group and AAV‐vehicle group (*n* = 3/group). (B) Representative double immunofluorescent staining of SLC7A11 and NeuN (*n* = 3/group). Statistical analysis was performed using one‐way ANOVA followed by Bonferroni correction for the multiple comparisons. *p < 0.05 versus control, ^#^ *p* < 0.05 versus AAV‐vehicle, ns indicates nonsignificant. Data are presented as mean ± SEM.

## Data Availability

The data that support the findings of this study are available from the corresponding author upon reasonable request. The Supporting Information is available in the supplementary section.
