# Supplementary figures and images for: Ferroptosis‐Driven Neuronal Damage Exacerbation in Diabetic Stroke: Implications of SLC7A11 Inhibition
Source: CNS Neurosci Ther. 2026 Mar 12;32(3):e70830. doi: 10.1002/cns.70830 (PMC13093281; doi:10.1002/cns.70830)

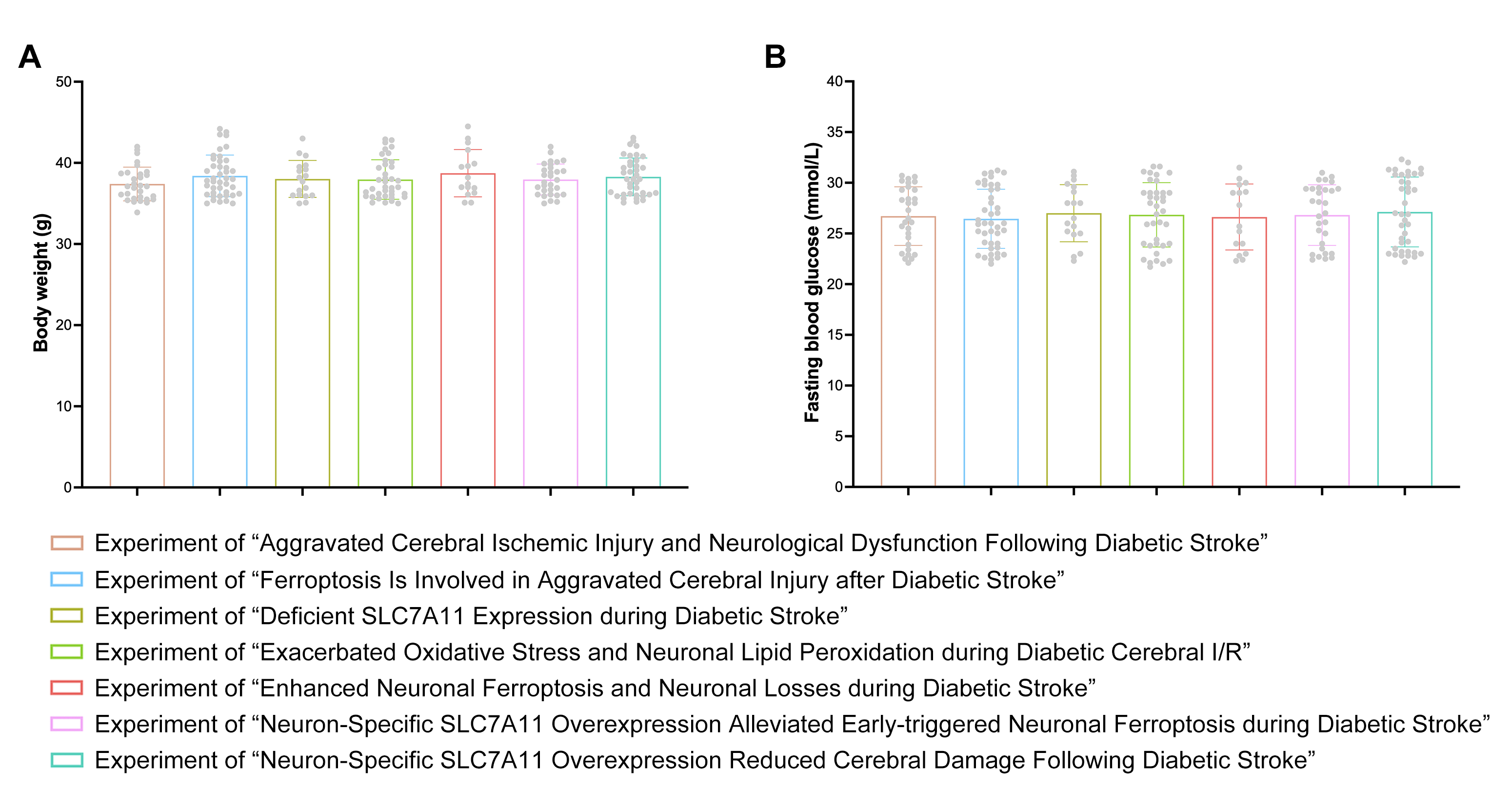

Supplement: Supplementary file 1 — Figure S1: Body weight and fasting blood glucose in db/db mice. (A) Body weight in db/db mice; (B) Fasting blood glucose in db/db mice. [file CNS-32-e70830-s001.tif]

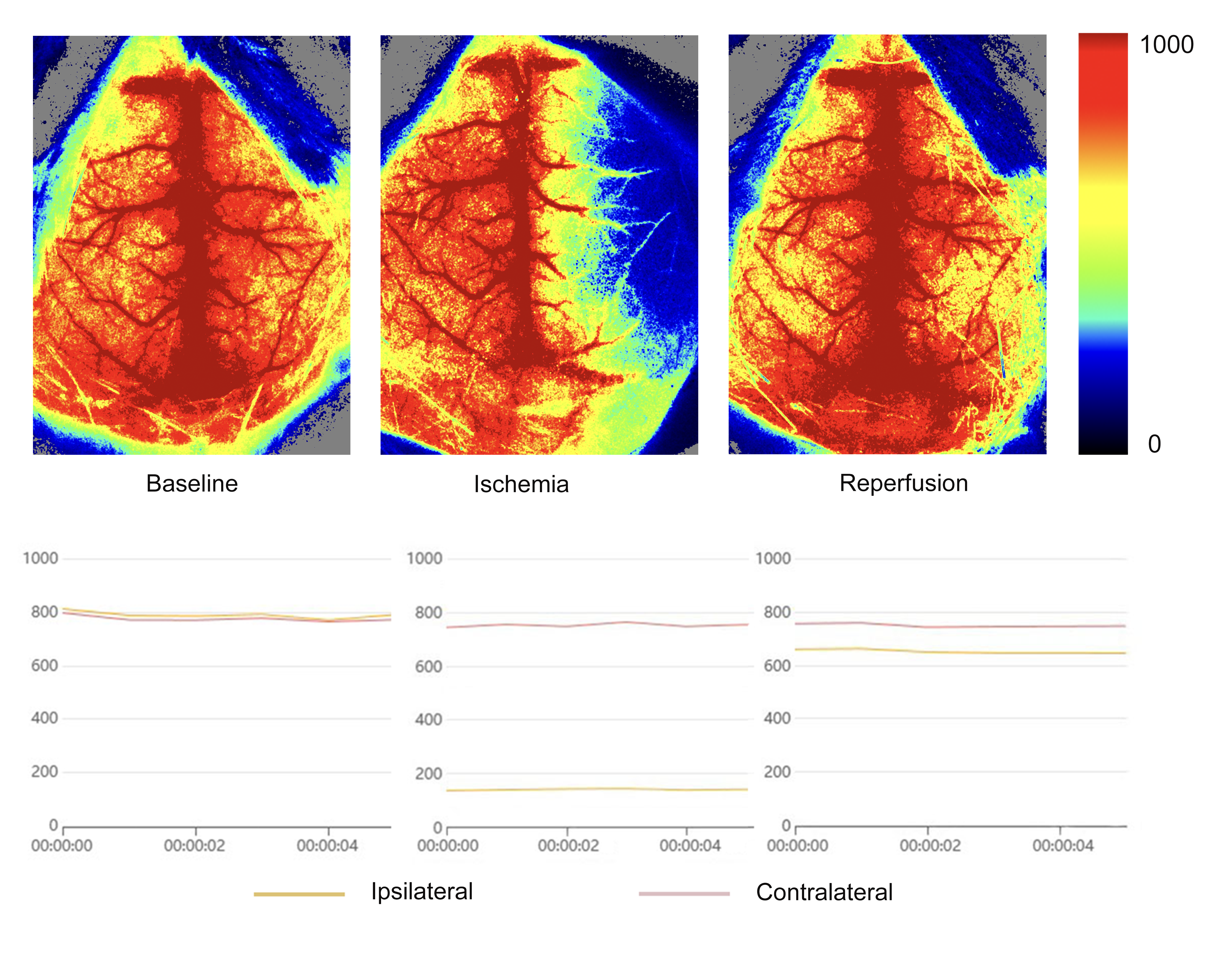

Supplement: Supplementary file 2 — Figure S2: Representative images of laser speckle flowmetry. Only the mice with at least 75% decline of cortical cerebral blood flow and 75% recovery of the baseline after reperfusion were included for the study. [file CNS-32-e70830-s003.tif]

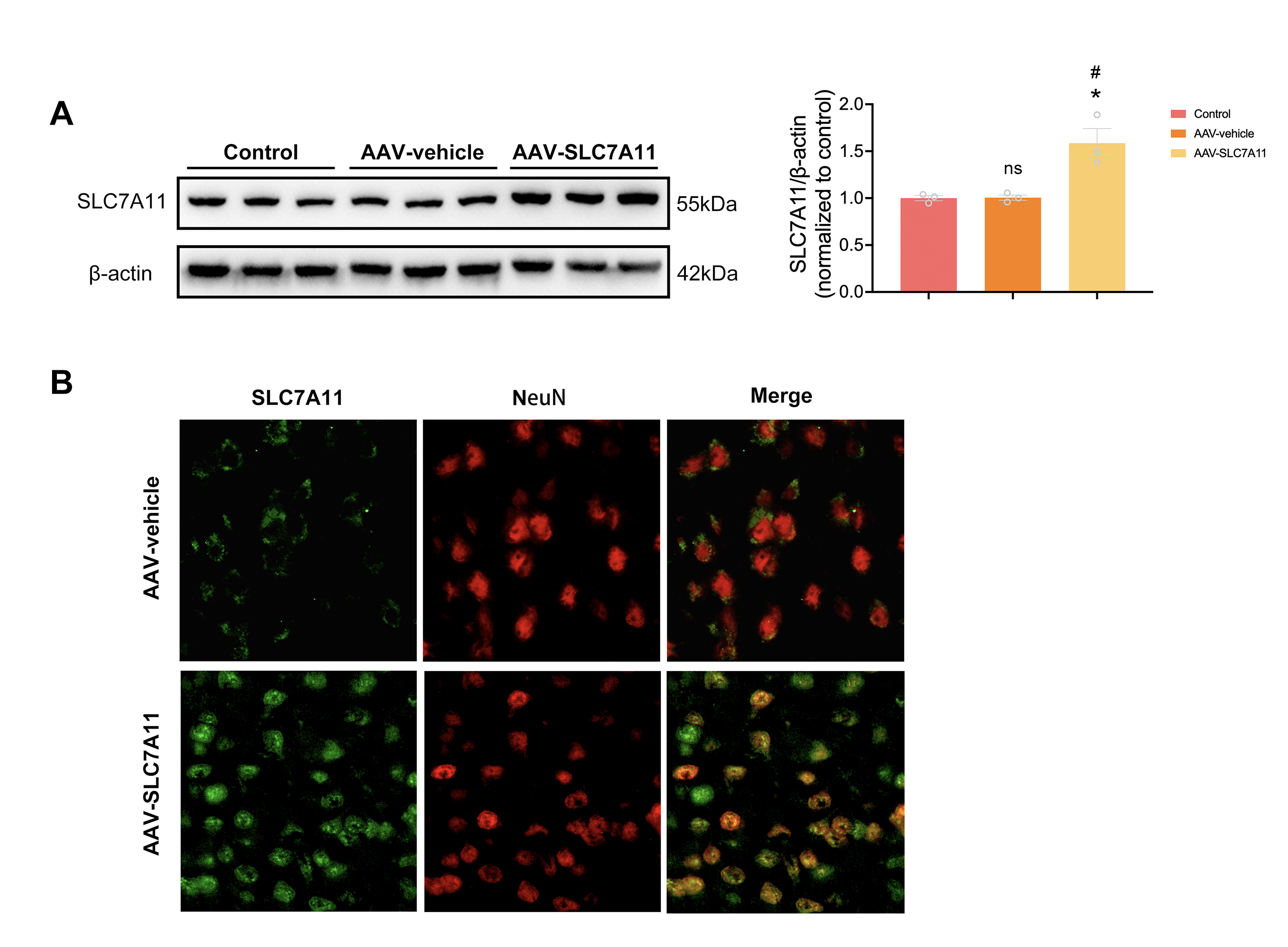

Supplement: Supplementary file 3 — Figure S3: Representative immunoblot and immunofluorescent staining of SLC7A11 protein expression. (A) Representative immunoblot and quantification of SLC7A11 protein levels at 4 h post‐reperfusion in the penumbra, β‐actin was used as internal control. SLC7A11 was upregulated in the AAV‐SLC7A11 group compared to the control group and AAV‐vehicle group (n = 3/group). (B) Representative double immunofluorescent staining of SLC7A11 and NeuN (n = 3/group). Statistical analysis was performed using one‐way ANOVA followed by Bonferroni correction for the multiple comparisons. *p < 0.05 versus control, # p < 0.05 versus AAV‐vehicle, ns indicates nonsignificant. Data are presented as mean ± SEM. [file CNS-32-e70830-s002.tif]
